# Periscope of Dermatology

**Published:** 1898-06-04

**Authors:** 


### Periscope of Dermatology.

(*Continued from page 152.*)

**Lupus.**—Finsen[18] uses concentrated red light in the treatment of lupus vulgaris. The action of light as a disinfectant and bactericide has already been recognised, especially with reference to tubercle bacilli. Now Finsen, by means of a simple apparatus, has concentrated the rays, either sunrays or electric arc rays, on patches of lupus, and in most cases has obtained satisfactory results. The rays at the violet end of the spectrum ("chemical rays") penetrate more deeply than do rays in other parts of the spectrum, and the almost translucent material of lupus nodules is more suited to transmit them than the ordinary skin. By this method of treatment, the chemical rays actually reach the tubercle bacilli, which cause the cutaneous affection, and gradually kill them if the lupus granulations be not too opaque.

Crocker[19] reports two cases of lupus erythematosus in which salicin had been administered in 15 grain doses three times a day, with excellent result, a cure occurring in one and great improvement in the other. The external treatment consisted in the application of calamine lotion, which was painted on twice a day. In the case in which a cure had resulted the treatment had been continued about five months; the second case had been under treatment for a shorter time. It was not claimed that salicin would cure every case, but that a good many were cured or improved by this form of treatment.

**Therapeutics.**—Cantrell[20] points out that arsenic has a rather limited field of usefulness in dermatology, and the drug may produce harm if improperly advised. Applied externally, it destroys abnormal tissue such as epithelioma, lupus, and hypertrophies; it, however, produces inflammation. Internally, it is most useful in pemphigus, acne rosacea, psoriasis, and lichen; in other diseases it is not of much use.

Paschkis[21] recommends xeroform in eczema and other conditions of the skin, in which there is a breach of surface, such as ulcers and burns. After cleansing the surface the powder is lightly dusted on, and when necessary a light gauze dressing applied. Superficial ulcers are healed in a few days, and dirty ones clean rapidly. It is a compound of bismuth and tri-bromo-phenol, and has no odour. It has no definite antiseptic properties, but favours healthy granulation and cicatrization.

Towsey[22] recommends the hypodermic injection of a 1 in 10 solution of thiosinamine in water in the treatment of keloid and inoperable cancer. The drug has no deleterious effects of any kind if not more than 15mm. be injected at a time. A considerable number of injections have to be made.

**Dermato-neuroses.**—Van Harlingen,[23] who has formerly collated and classified the various skin manifestations met with in the hysterical condition, now reports five cases in females for which he suggests the name "Dermatoneurosis erythematosa hysterica," coinciding in their main features with those described, under the title of "Neurotic Excoriations," by Erasmus Wilson. Van Harlingen feels confident that they are dermato-neuroses, and not factitious affections of the skin.

Savill[24] reports two lectures on dermato-neuroses, in which he includes prurigo, chloasma, wrinkles, Raynaud's disease, acroparæsthesia and fugitive erythema. He urges the importance of treatment by galvanism.

Feindel[25] refers to a condition called general neuro-fibromatosis, which is characterised by the existence of tumours, pigmentation of the skin, mental phenomena, and alteration of sensation and of movements. The tumours, which are both cutaneous and connected with the nerves, occur anywhere except on the face and the palms and soles. The pigmentation spots are either small or involve large areas. There is anæsthesia, cramp, and loss of memory. The disease is generally congenital, but it may lay dormant for years; it gradually progresses, and patients die in a condition of extreme marasmus.

[18] Brit. Med. Jour. Epit. Jan. 29, 1898, No. 90. [19] Brit. Jour. of Derm., Jan. 1898, and Amer. Jour. Med. Sci., Mar. 1898, p. 366. [20] Med. Chron., Feb. 1898, p. 351. [21] Brit. Med. Jour. Ep., Jan. 22, 1898, No. 71. [22] New York Med. Jour., Nov., 1897, p. 624. [23] Int. Med. Mag., Nov. 1897, p. 695, and Brit. Journ. of Derm , Mar. 1898, p. 106. [24] Clin. Journ., Mar. 2, 1898, p. 317, and Mar. 9, 1898, p. 345. [25] Brit. Med. Jour. Epit., Dec. 25, 1897, No. 522.

